# Expectancy-Value Model Related to Physical Activity Behaviors in Chilean and Spanish Adolescents

**DOI:** 10.3390/ijerph17218219

**Published:** 2020-11-06

**Authors:** Laura O. Gallardo, Alberto Abarca-Sos, Alberto Moreno Doña

**Affiliations:** 1 Faculty of Social Sciences and Humanities, University of Zaragoza, 44003 Teruel, Spain; lau@unizar.es; 2 Faculty of Medicine, Universidad de Valparaíso, 2520000 Viña del Mar, Chile; alberto.moreno@uv.cl

**Keywords:** physical activity, intention to be physically active, teasing, competence, appearance, stress, enjoyment, friend support

## Abstract

The purpose of the study is to comparatively test the expectancy-value model in Chilean and Spanish samples. The model proposes: a social world (composed of social support, physical activity teasing, and weight teasing), expectancy (composed of perceived competence and appearance), task values (composed of enjoyment and stress) to predict physical activity and intention to be physically active. Participants were 497 (Chilean) and 1365 (Spanish) adolescents. Structural equation models and multi-group modelling were used. All the models presented adequate fit to the data. The results show that physical activity teasing is a contextual and essential variable; perceived competence and enjoyment influenced physical activity and intentions to be physically active; some differences appeared in the prediction of physical activity and intentions to be physically active when the multi-group model was run. Culturally tailored interventions are key to improving physical activity (PA) behaviors.

## 1. Introduction

Physical inactivity is currently the fourth leading cause of death worldwide, and one of the most important determinants of chronic diseases such as type II diabetes, colon cancer or osteoporosis (World Health Organization) [1]. In recent studies, the proportion of young people who do not perform some physical activity (PA) is high [2]. Aubert et al. [3], in a large global study conducted in 49 countries with young people aged 5 to 17, indicated that PA levels had a “low/poor” average value, which corresponds to between 27% and 33% of compliance with the recommendations for children and adolescents. Gender and age have shown consistent results concerning PA, with boys performing more PA than girls [4] and PA declining during adolescence [5]. Thus, these personal variables should be measured to control their effects.

Adolescence is a critical period because there are certain cognitive (way of thinking), psychological (identity building) and social changes (new friendships, family relationships) in young people [6]. Because of these changes, much research has focused on studying PA concerning the intention to be physically active in the future [7]. Intentions can predict behavior linked to PA practice in adulthood, and it may be useful to identify their determinants, as a strategy for reducing physical inactivity [8].

Thus, to implement successful interventions, the identification of influential factors of PA levels and intentions is essential. Previous studies have used the expectancy-value (EV) model to address this issue [9,10]. This framework provides two important predictors of choice behaviors: first, individuals’ expectations of success, and, second, the subjective task value [9,10]. Thus, factors such as self-beliefs and affective memories are significantly related to adolescents’ time spent performing PA [11].

Firstly, concerning self-beliefs, perceived competence and appearance have been related to PA, acting as positive and significant determinants [12]. Perceived competence has been defined by Harter [13] as children’s self-evaluative judgment about their ability to accomplish certain tasks in a given domain, in our case, PA. Appearance is related to an individuals’ assessment of their body and/or shape [14]. Both variables, integrated into the self-concept construct [14], play an essential role in adolescent PA. Perceived competence is the variable most strongly associated with PA [12], and appearance has been related to PA levels, as well to as other health variables such as better eating habits and lower probability of suffering overweight [15].

Secondly, the EV component task value refers to the importance and interest attributed to PA, related to affective memories. Four components of values have been defined by Wigfield and Eccles [16]: intrinsic value (enjoyment of PA), utility value (usefulness of PA related to individuals’ present or future goals), attainment value (importance of doing PA well), and cost value (perceived negative aspects of engaging in PA). Related to these four components, two variables have been consistently related to PA: enjoyment and stress [17,18]. Enjoyment, related to intrinsic value, has been defined as the state or process of taking pleasure in something [19]. When PA interventions were carried out with enjoyment, the experimental group scored significantly higher than the control group, which led to increases in PA levels [20]. Stress, related to cost value, has been described as “the negative feelings and beliefs that people experience when they feel they cannot cope with the demands of their environment” [21]. Stress in adolescents can be provoked by several demands, such as managing their peers’ or parents’ expectations, academic achievement, or PA participation [22].

Summing up, the EV model provides expectancy variables—perceived competence and appearance—and task value variables—enjoyment and stress—as predictors of PA [9,10]. However, the EV model also indicates that adolescents’ social world, such as past events, influences their expectancies and task values [9].

A relevant aspect in adolescence is that young people receive negative verbal feedback or weight-related teasing [23]. Although teasing has been linked to health-related variables, there are very few studies that link PA to weight and PA teasing, and the conclusions are weak. For instance, two studies found a significant and negative relationships [24,25]. Another study recently found no significant relationships between different PA intensities and weight teasing during PA [26]. However, different dimensions of physical self-concept, such as competence or appearance, are strongly and significantly related to PA [27]. Thus, weight and PA teasing seem to be important variables to consider in adolescents’ social context. Moreover, teasing can affect girls and boys differently in the PA and sport context because weight teasing is received both by males and females, whereas PA teasing is only perceived by male adolescents [28]. Furthermore, obese and overweight youth report high levels of weight teasing [29]. Thus, body mass index (BMI) is an important variable to control for when teasing is analyzed in adolescents. Moreover, at the social level, the EV model emphasizes the role of friends’ influence in self- perceptions related to PA [17]. Previous studies have addressed this issue consistently [30].

PA and its influential variables can vary in populations from different countries [31]. Recent studies show that, in European and American countries, PA varies depending on socioeconomic status [32]. Moreover, in a study with 1.6 million adolescents in 146 countries, it was found that the most inactive participants were boys living in Western countries and girls from South Asian countries [33]. Specifically, in Latin American countries, results revealed similar patterns, with significant differences in PA and different determinants [34,35]. Because of these differences, some authors point out the importance of making international comparisons, as they may illustrate relevant patterns and trends of PA practice [31].

**Figure 1:**
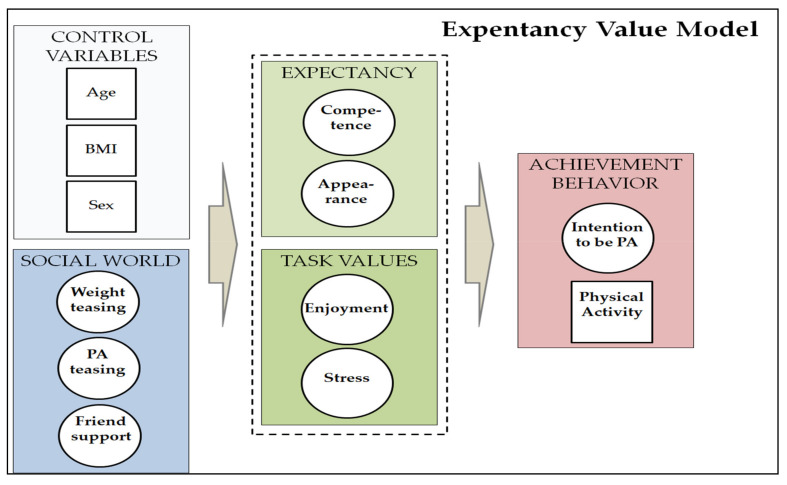
Explanatory model of physical activity behavior and intention to be physically active, adapted from Eccles et al. [9].

In the previous literature, we have not found any studies that integrated the EV model into PA and the intention to be physically active in a cultural comparison by country. Understanding how the social world may influence expectancy and task values and subsequently influence behavior (Figure 1), analyzed by country, will allow PA promotors and professionals to individualize interventions by region or country, focusing on specific variables that influence behavior and intentions.

Thus, the aims of the study are: (1) to examine the EV model to determine whether the social world (age, sex, BMI, friends’ support, weight teasing, and PA teasing) influences individuals’ expectancy (competence and appearance) and task values (enjoyment and stress), and hence their achievement behavior (PA and the intention to be physically active); (2) to analyze the differences in the EV model by country, Chile and Spain; (3) to assess teasing differences (weight and PA teasing).

Concerning these goals, it was hypothesized that: (a) weight and PA teasing would negatively influence perceived competence, appearance, and enjoyment, and they would positively influence stress; (b) friend support would positively influence perceived competence, appearance, and enjoyment, and it would negatively influence stress; (c) perceived competence, appearance, and enjoyment would positively affect PA and intention to be physically active, and stress would affect them negatively; (d) country differences in the paths would be found when conducting multi-group analysis; (e) weight and PA teasing would act similarly in Chilean and Spanish samples.

## 2. Materials and Methods

### 2.1. Sample

Participants (*N* = 1862) were students from 1st grade through 4th grade of secondary education (equivalent to 7th–10th grade in the USA) recruited in the northeast of Spain (*N* = 1365; *M_age_* = 14.5, *SD* = 1.36; 49.1% females) and the northwest of Chile (*N* = 497; *M_age_* = 14.7, *SD* = 1.47; 54.2% females). The inclusion criteria were that participants had completed all the measures. A total of 14 Spanish and six Chilean secondary schools participated in the study. No students or their guardians refused to participate in this study. T-tests were conducted to compare the above-mentioned original (*N_Spain_* = 2220; *N_Chile_* = 734) and final samples, finding no significant differences between participants who remained in the sample and those who withdrew from the study (*p* > 0.05).

All subjects gave their informed consent for inclusion before they participated in the study. The study was conducted in accordance with the Declaration of Helsinki, and the protocol was approved by the Clinical Research Ethics Committee of Aragon (Spain) in the 29 April 2015 (PI15/0063).

### 2.2. Measurement Instruments

International Physical Activity Questionnaire short form (IPAQ-SF) [36]. The version used was adapted to Spanish adolescents by Aibar et al. [37]. The questionnaire has showed significant and positive correlations with MTI Actigraph accelerometers (moderate to vigorous PA, *r* = 0.55, *p* < 0.01; moderate PA, *r* = 0.37, *p* < 0.05; vigorous PA, *r* = 0.34, *p* < 0.05). The IPAQ-SF estimates the daily minutes of vigorous and moderate PA that adolescents practice daily. The questionnaire is composed of 7 items (e.g., During the last 7 days, on how many days did you do vigorous physical activities like heavy lifting, digging, aerobics, or fast bicycling?). PA was calculated using the vigorous and moderate items, multiplying the minutes of PA by the number of days and adding the two intensities (Craig et al., 2013).

Intention to be Physically Active Scale [38]. The Spanish version found an adequate internal consistency, α = 0.94 [39]. The instrument consists of 5 items (e.g., “*After graduation, I would like to be physically active*”), rated on a five-point Likert scale ranging from 1 (completely disagree) to 5 (completely agree). *Intention to be physically active was estimated with the items’ arithmetic mean as the authors recommended*.

Physical Self Description Questionnaire (PSDQ) [40]. The PSDQ is a multidimensional questionnaire designed to measure several domains of physical self-concept. In this study, we used Perceived Competence and Appearance. The Spanish version was validated in adolescents by Marsh, Marco, and Abcy [41], showing good internal consistency (alphas between 0.82 and 0.91). The chosen scale variables were comprised of 6 items each one rated on a six-point Likert scale ranging from 1 (false) to 6 (true). An example item of Perceived Competence is “I am good at most sports and doing physical activity”, and of Appearance, “I look good“.

Physical Activity and Sport Enjoyment Satisfaction Scale [19]. This questionnaire was validated and adapted to Spanish by Cervelló, Escartí and Balaguer [42], showing an internal consistency of α = 0.74. The instrument consists of 5 items (e.g., *“I usually have fun doing physical activity and sports”*) and is rated on a five-point Likert scale ranging from 1 (completely disagree) to 5 (completely agree).

Perceived Stress Scale (PSS) [43]. The PSS assesses how often an adolescent has experienced stress over the past month. The Spanish version was validated by Remor and Carrobles [44], finding a Cronbach alpha of α = 0.81. The original scale has 14 items, but the shorter 4-item version of the PSS, with appropriate psychometric properties [45], was used in the present study. An example item is “How often have you felt nervous and stressed when you practiced PA?” Items are rated on a five-point Likert scale ranging from 0 (never) to 4 (always).

Perception of Weight-Related Teasing and PA teasing (POTS) [46]. The POTS measures an individuals’ history of being teased about weight. Each subscale consists of 6 items to assess both the frequency of teasing—ranging from 1 (never) to 5 (very often)—and its impact on the individual—ranging from 1 (not upset) 5 (very upset). The PA teasing scale was adapted to PA context. Example items are: “People made fun of you when you practiced physical activity, physical education, or sports” or “People pointed at you because you were overweight.” Higher scores denote greater weight and PA teasing exposure and sensitivity. The validated Spanish version of POTS [47] had an internal consistency ranging between 0.89 and 0.95.

Sport Friendship Quality Scale (SFQS) [48]. The SFQS is composed of 5 dimensions. In this study, only the Self-Esteem Enhancement and Supportiveness dimension was used to assess sport friendship quality, because it is related to self-concept and social support. The scale is made up of 4 items (e.g., “My friend has confidence in me during sports”) rated on a five-point Likert scale, ranging from 1 (not at all true) to 5 (really true). This questionnaire was used with Spanish adolescents, yielding an adequate internal consistency, α = 0.81 [49].

Socio-Demographic Variables. We recorded participants’ general characteristics: sex, age, and body mass index (BMI). Participants reported their sex and age with a single question. They were also asked to provide their weight in kilograms and height in meters to calculate BMI with the following formula: weight(kg)/height(m^2^). Self-reported weight and height showed positive and significant correlations with measured weight and height. Thus, self-reported BMI can be used as a simple and valid tool for BMI estimates of overweight and obesity in epidemiological studies [50].

### 2.3. Procedure

Data collection was carried out during spring in Spain and fall in Chile, following the same procedure for Spain and Chile. Firstly, the researchers contacted the education authority and management teams of the secondary schools in Chile and Spain. Secondly, an informative circular was given to participants, including informed consent for parents/guardians. A consent form for the participants to be signed was included in the questionnaire. Thirdly, a member of the research team administered the questionnaire during the students’ regular school classes. The questionnaire was adapted to the Chilean sample as follows below. To ensure that there was no bias due to reading difficulties, at least one researcher remained in the room to monitor students’ progress and answer any questions. Before completing the questionnaire, we informed the students that all of their answers were confidential and their participation was voluntary.

The adaptation to the Chilean sample was developed following the cultural translation performed by Sousa and Rojjanasrirat [51]. Firstly, two reviewers from Chile, whose native language was Chilean Spanish, carried out reviews of the questionnaire independently. Then, translations T1 and T2 were obtained. Secondly, a meeting was held between the research team and the translators to obtain an initial version by consensus. The adaptation was conducted in two facets: the first one was a simple semantic adaptation, and the second one a conceptual translation to simplify the expressions for adolescents. Thirdly, a pilot test was carried with a small sample of 12- to 16-year-old adolescents to obtain the final version.

### 2.4. Data Analysis

Data were analyzed with Mplus, Version 7.11 to implement SEM models and multi-group SEM models by country. All the models are composed of 8 latent variables and 3 exogenous variables. PA was the exception, which was calculated following the questionnaire validation protocol [36]. To be able to compare the groups, the assumption that the instruments measure the same psychological construct at all times and in all groups must be met. If it is met, the comparisons are valid and differences/similarities/variations between groups can be meaningfully interpreted [52]. Hence, measurement invariance was tested. For this purpose, an unconstrained model was established and hierarchically advanced to more restricted (and nested) models [53]. The invariance routine started by testing the unconstrained model, in which the pattern of indicator-to-construct is equal across groups (configural invariance). This baseline model was subsequently compared with the next level of measurement invariance, including factor loading equality (weak factorial invariance), equality of the intercepts of the corresponding indicators (strong factorial invariance), and equality of the residual variances of the corresponding indicators (strict factorial invariance). In the models, the residuals of the corresponding indicators were allowed to correlate across groups, and the first factor loading per latent variable was set to the unity to establish the scale of latent variables, as recommended by Little, Preacher, Selig, and Card [54]. “A value of ΔCFI smaller than or equal to 0.01 indicates that the null hypothesis of invariance should not be rejected” [55].

An SEM model and a multi-group model were analyzed in the following sequence. The models assessed whether the social world (age, sex, BMI, Friend Support, Weight teasing, and PA teasing) influence individuals’ expectancy (Competence and Appearance) and task values (Enjoyment and Stress) and, therefore, PA and the intention to be physically active. The multi-group model added country belongingness (Chile and Spain). Although a multi-group SEM model does not demonstrate causality [56], this approach allows for exploring and testing key issues in terms of the pattern of relations among groups. Finally, nested models were developed to determine whether teasing acts similarly in Chilean and Spanish samples.

Considering the possible multivariate non-normality of the measures, the robust maximum likelihood (MLR) estimator was selected for model estimations [57]. Goodness of fit was tested with common fit indexes. Thus, a model fit is considered adequate when the Comparative Fit Index (CFI) and the Tucker–Lewis Index (TLI) have values > 0.90, the Root Mean Square Error of Approximation (RMSEA) is < 0.06, and the Standardized Root Mean Square Residual (SRMR) is < 0.08 [58].

## 3. Results

Descriptive statistics and the correlation matrix between the model variables are shown in Table 1. The table also contains reliabilities of the latent variables for the Chilean and Spanish questionnaires, showing good results in both samples.

To highlight the correlation results, the dependent variables (intentions to be physically active and PA) were related to the rest of the target variables in the Spanish sample, except for age. Intention to be physically active in the Chilean sample was not related to weight or PA teasing. Moreover, in the Chilean sample, PA was not related to weight or PA teasing, Appearance, Stress, or BMI.

### 3.1. Previous Analysis: Invariance Testing

Tests of measurement invariance are presented in Table 2. A decrease in CFI < 0.01 implies invariance. Thus, according to this criterion, weak and strong measurement invariance was supported in the group comparisons. The most parsimonious model, both with the invariance of factor loadings and item intercepts across groups, was selected. This implies that the measures across groups are considered to be on the same scale (equality of factor loadings) and the item scores of the different groups have the same measurement metric and the same scalar, allowing for the comparison across groups of the means of the underlying factors. Consequently, substantive across-group comparisons can be made [59].

### 3.2. The Overall Model

The overall model with the entire sample presented an adequate fit to the data, *χ*^2^(904) = 3058.329, CFI = 0.942, TLI = 0.937, RMSEA = 0.036, 90% CI [0.035, 0.038], SRMR = 0.041. Focusing on the structural model paths, the standardized parameters were significant: Competence predicted PA (β = 0.41, *p* < 0.001) and intentions to be physically active (β = 0.50, *p* < 0.001); Enjoyment predicted PA (β = 0.08, *p* < 0.001) and intentions to be physically active (β = 0.42, *p* < 0.001); PA teasing predicted Competence (β = −0.15, *p* < 0.001), Appearance (β = −0.14, *p* < 0.001), Enjoyment (β = −0.10, *p* < 0.01), and Stress (β = 0.33, *p* < 0.001); Friend Support predicted Competence (β = 0.40, *p* < 0.001), Appearance (β = 0.26, *p* < 0.001), (β = 0.40, *p* < 0.001), and Enjoyment (β = 0.47, *p* < 0.001); Age predicted Competence (β = −0.05, *p* < 0.05) and Enjoyment (β = −0.09, *p* < 0.001); BMI predicted Competence (β = −0.12, *p* < 0.001) and Appearance (β = −0.11, *p* < 0.001); Sex predicted Competence (β = −0.20, *p* < 0.001), Appearance (β = −0.08, *p* < 0.001), Enjoyment (β = −0.13, *p* < 0.001), and Stress (β = 0.06, *p* < 0.05). Perceived Competence was related to Appearance (*r* = 0.42, *p* < 0.001), Enjoyment (*r* = 0.45, *p* < 0.001), and Stress (*r* = −0.24, *p* < 0.001); Appearance was related to Enjoyment (*r* = 0.18, *p* < 0.001) and Stress (*r* = −0.12, *p* < 0.001). Enjoyment and Stress were correlated (*r* = −0.21, *p* < 0.001). Lastly, PA and intention to be physically active also correlated (*r* = 0.28, *p* < 0.001).

### 3.3. Comparing Models: Multi-Group Analysis by Country

**Figure 2:**
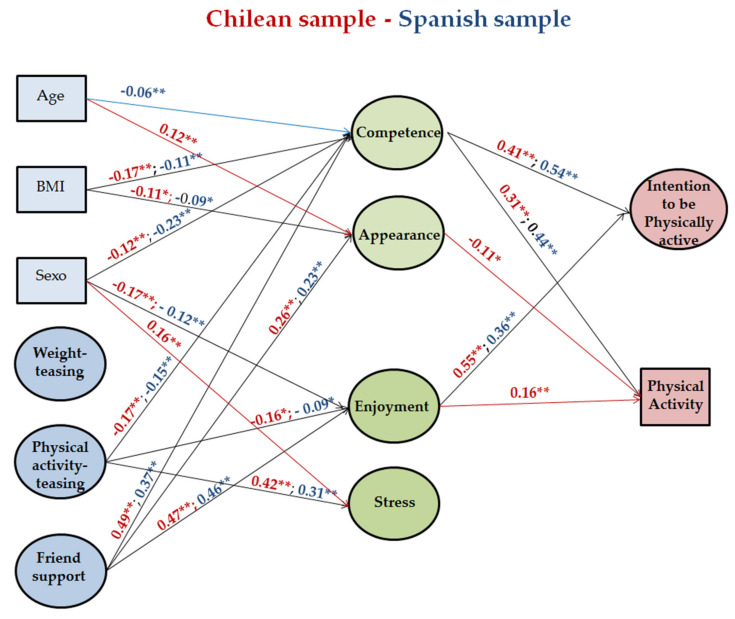
The multi-group final model where standardized parameters of the paths between countries can be compared. Covariances, correlations, and nonsignificant paths are omitted for presentation clarity. Chilean results are in red, Spanish results are in blue. ** *p* < 0.01, * *p* < 0.05.

Once measurement invariance was obtained, multi-group analysis was run. Figure 2 presents all the standardized parameters examining the relations in the proposed EV model. The model presented an adequate fit to the data, χ^2^(2056) = 4810.658, CFI = 0.931, TLI = 0.927, RMSEA = 0.039, 90% CI [0.037, 0.040], SRMR = 0.051.

The main differences between the countries are in the predictors of PA. In the Spanish sample, only Competence was a predictor whereas, in the Chilean sample, Competence, Appearance, and Enjoyment predicted PA. Moreover, age and sex behaved differently, the two control variables were only significant in the Chilean sample, as shown in Figure 2.

Correlation results showed that Perceived Competence was related to Appearance (*r* = 0.36, *p* < 0.001), Enjoyment (*r* = 0.44, *p* < 0.001), and Stress (*r* =−0.19, *p* < 0.001); Enjoyment and Stress were correlated (*r* = −0.24, *p* < 0.001). Finally, PA and intention to be physically active were also correlated (*r* = 0.32, *p* < 0.001).

### 3.4. Testing Teasing Differences

To test teasing differences, firstly, we conducted an independent-sample t-test by sex. Only the Chilean sample showed a difference in PA teasing, where boys scored higher than girls in PA teasing t_663_ = 1.99, *p* = 0.04.

Additionally, the invariance of the structural path coefficients of the teasing variables was tested across groups. We wondered whether teasing acts similarly in Chilean and Spanish samples. For this purpose, firstly, we estimated a configural SEM model using the Chilean and Spanish samples simultaneously. This model served as a base model for model comparisons. All path coefficients in the model were set free across groups. Subsequently, we imposed restrictions to be estimated equally across groups in the path coefficients one-by-one and compared to the base model. The restrictions were, first, in the path coefficient between Weight teasing and Competence, then between Weight teasing and Appearance, and so on. The results of the Weight teasing restrictions show no significant difference. Statistically speaking, population membership (Chilean or Spanish) did not significantly moderate the effect of Weight teasing on expectancy or value variables. The effect remains invariant among country membership. The results of the PA teasing restrictions show no significant difference in country membership in the paths of PA teasing and Competence, PA teasing and Appearance, and PA teasing and Enjoyment. In contrast, the path coefficient between PA teasing and Stress was significant, χ^2^(3) = 25.819, *p* < 0.001). This means that country moderates the effect of PA teasing on Stress among adolescents.

## 4. Discussion

This study sought to test the EV model in adolescent samples from Chile and Spain. The model fits the data, and we found differences by country. Innovative social variables were deeply studied, compared, as well as effects such as Weight and PA teasing. Some important findings are shown with implications for interventions. Next, each hypothesis is discussed, and conclusions and future research are suggested.

Considering the proposed model, firstly, we hypothesized that weight and PA teasing would negatively influence perceived competence, appearance, and enjoyment, whereas they would positively influence stress. Our results showed that, indeed, PA teasing negatively influenced perceived competence, appearance, and enjoyment and positively influenced stress, and this was statistically significant. On the contrary, weight teasing did not influence any of the expectancy or task value variables. The scarce research that relates weight teasing and diverse PA behaviors and attitudes has found negative relations between being weight-teased and PA self-efficacy, physical self-concept, enjoyment, or PA behaviors [60,61,62]. Nevertheless, none of them have previously assessed PA teasing and weight teasing at the same time. Our results indicate that the influence of being teased is a specific domain. Thus, although weight teasing may influence PA attitudes or behaviors, when both teasing experiences are included, only PA teasing appears to influence the predictors of PA behaviors, when controlling for BMI, sex, and age variables. PA teasing is a specific and essential measure related to the area of PA, which has been shown to influence expectancy and task value variables as predictors of PA achievement behaviors.

Continuing with the social world, the second hypothesis indicated that friend support would positively influence perceived competence, appearance, and enjoyment, and negatively influence stress. The results partially supported the hypothesis because the relationships found have shown that friend support is significantly and positively related to competence, appearance, and enjoyment, whereas it had no significant association with stress. These findings are in line with previous results [30,63], because there is a strong association between PA and friend support, although the relationship is mediated by psychological variables [64]. Specifically, the scientific literature has shown positive associations between friend support and competence [49], appearance [65], and enjoyment [17]. However, concerning stress, the lack of relationships with PA could be explained because it may not be linear. Moljord et al. [66] found that adolescents who performed PA two or three times a week suffered less stress than youth who performed PA one day or four or more days. Therefore, friend support emerges as a relevant determinant in the EV model and confirms that adolescents are sensitive to this social agent.

The third hypothesis, stating that perceived competence, appearance, and enjoyment would positively affect PA and negatively affect stress and intention to be physically active. Our results indicate that perceived competence and enjoyment were positively related to PA and intention to be physically active, whereas appearance and stress were not significantly related to either behavioral variable. The hypothesis is only partially supported because no appearance and stress relationships were found.

Firstly, perceived competence and enjoyment are both important to understand the intention to be physically active and PA, consistently with the EV model of adolescent PA behavior [10]. In a recent study, perceived competence and enjoyment were strongly related to PA, as in our study [67]. These results could be explained because adolescents who report stronger perceived competence are more likely to enjoy PA, thus continuing to engage in the behavior for a long time [68].

Secondly, appearance was not related to PA and intention to be physically active. Appearance and perceived competence were supposed to be predictors of PA, mediating the effects of the social world. However, only the latter was significantly related to PA. Similar results were reported by Raudsepp et al. [69], who found that the best predictor of PA and physical fitness was perceived competence, not appearance. These results may be explained by sex differences in perceptions because previous research has corroborated that girls’ perceived appearance was lower than boys’ [40,70]. In our study, no sex differences in the paths were recorded. Thus, significant relationships may change when boys’ and girls’ relationships are analyzed separately. Thus, current evidence does not support a clear association between appearance and PA in adolescents. More research is needed to clarify this relationship.

Thirdly, stress was not related to PA and intention to be physically active. These results were not in line with the EV model and studies that have analyzed these relationships [18]. However, other studies have also reached inconclusive results. For instance, Smith Carter [71], in a longitudinal study, found that stress predicts PA in one out of the three measurement times. Moreover, in our study, stress was predicted by PA teasing, so the relationship between stress and PA should be analyzed in future studies because of their inconsistent relationship. In our study, the lack of relationships between PA and intention to be physically active could be explained because other variables of the model, such as enjoyment and perceived competence, are strongly and consistently related [67], leading to this lack of effect.

Regarding the multi-group analysis by country, the fourth hypothesis stated that multi-group analysis would reveal differences in the paths by country. This hypothesis was corroborated. The main differences appear in the PA predictors, because, in the Spanish sample, competence is significantly and positively related to PA, whereas, in the Chilean sample, competence, appearance (negatively) and enjoyment predicted PA. Moreover, sex and age were only significantly related to stress and appearance in the Chilean sample. To understand country differences in PA, several factors, such as the Human Development Index, the per capita income, or income inequality should be studied [31]. Following the last publication of the United Nations Development Programme [72], Chile and Spain are categorized as having very high human development, but the per capita income shows differences: Chile USD 21,972.27 and Spain USD 35,041.29. However, different studies have shown that PA levels are high in higher-income households of high-income countries and lower in higher-income households of low–medium-income countries.

Therefore, other variables related to countries’ cultures may influence the results. In the present study, most relationships were found to be universal across Chile and Spain. However, enjoyment was only positively related to PA in Chile. This result could be explained by the countries’ differences in organized PA and physical education. In a comparative study, Chilean adolescents were less pleased with the physical education they received than German adolescents [73], a variable related to enjoyment. Thus, physical education programs developed in Chile could influence PA whereas, in European countries, these programs are more homogeneous and enjoyment has no effect on PA.

Appearance negatively influences PA and positive relationships between age and appearance could be explained by differences in culture because body satisfaction changes depending on race or working class [74,75]. Thus, regarding this fluctuation in self-perceptions as a function of culture, the paths and influence could vary. Concerning this finding, sex influences stress in the Chilean population, such that girls perceived more stress than boys. This could be explained by culture because, in several studies, sex differences in stress and their relationships with PA were non-significant [71,76].

Chile and Spain are similar in several cultural dimensions, whereas they present differences in others. Further research should address this topic. Therefore, culturally tailored interventions by country and culture should be implemented because they would be more likely to improve PA.

In the last hypothesis, we wondered whether weight teasing and PA teasing have similar effects in Chilean and Spanish samples. Our results show that only PA teasing presented sex differences in the Chilean sample. Boys scored higher than girls. Nevertheless, we found no previous research that examined sex differences in PA teasing behaviors. Weight teasing did not show sex differences in any sample, which agrees with the POTS validation [46], where the authors found no significant mean sex differences in the validation model. This means that weight teasing does not depend on sex. Additionally, the teasing variables were tested to determine whether they behave differently by country. Our results show that the effect of weight teasing and PA teasing remained invariant among countries, except for the effect of PA teasing on stress, which was moderated by country membership; that is, this relation will vary across countries. Further investigations are needed to examine stress results regarding PA teasing and PA behaviors.

It is important to bear in mind that our study only has two samples. Hence, prospective studies with more countries could support our results and determine new determinants in the EV model. Our study is based on cross-sectional data, which do not allow us to propose causal relations. Longitudinal data are very interesting to establish causal relations. Finally, we used self-reported questionnaires, which may be affected by subjective experiences and beliefs. Additional objective data, such as peer relations or accelerometry, may provide important information for the model.

## 5. Conclusions

The present study tested the EV model, showing good results with a sample composed of participants from two countries and also using multi-group SEM models by country. PA teasing, and not weight teasing, appears as a specific contextual variable related to PA behaviors. Therefore, in the educational and sport context, professionals will have to focus their attention on preventing PA teasing. More research is needed to understand how PA teasing and weight teasing influence PA behavior, because there is no consensus in the literature and the results found in this study are inconclusive. Friend support emerges as a relevant determinant in the EV model as part of the social world and confirms that PA behaviors in adolescents are sensitive to social agents. Perceived competence (as an expectancy variable) and enjoyment (as a task value variable) predict PA behaviors. Thus, teachers and coaches will have to prepare their programs to be fun and to encourage students to increase their perception of competence. When country membership is taken into account, some differences appear in the prediction of PA behaviors: sex, appearance and enjoyment are a determinant in the Chilean sample but not in the Spanish sample. In light of the results, culturally tailored interventions are essential to take into account the key variables to improve PA behaviors.

## Figures and Tables

**Table 1:** Means, standard deviations, reliabilities, and correlations between variables under study.

	Chile	*M*	*SD*	α	1	2	3	4	5	6	7	8	9	10	11	12
Spain																
***M***		–			7.45	8.82	3.80	23.74	23.67	4.12	5.21	3.78	39.22	14.70	22.44	–
***SD***			–		3.70	3.70	1.09	8.22	7.79	0.95	4.28	0.95	37.87	1.49	5.23	–
**α**				–	0.945	0.867	0.856	0.936	0.884	0.901	0.826	0.813	–	–	–	–
**1 Weight teasing**		7.92	4.63	0.957	–	**0.61**	−0.08	**−0.11**	−0.07	−0.05	**0.18**	−0.03	−0.02	0.05	**0.15**	−0.07
**2 PA teasing**		9.23	4.66	0.923	**0.61**	−	−0.07	**−0.10**	**−0.10**	−0.08	**0.30**	−0.02	0.05	−0.06	−0.05	**−0.11**
**3 Friend support**		4.02	0.93	0.842	**−0.10**	**−0.15**	**−**	**0.43**	**0.23**	**0.41**	−0.08	**0.41**	**0.17**	0.03	-0.02	**0.02**
**4 Competence**		24.10	7.16	0.932	**−0.18**	**−0.23**	**0.33**	**–**	**0.41**	**0.56**	**−0.23**	**0.60**	**0.34**	−0.05	**−0.19**	**−0.12**
**5 Appearance**		25.45	5.87	0.812	**−0.23**	**−0.27**	**0.21**	**0.46**	**−**	**0.18**	**−0.14**	**0.19**	0.05	**0.09**	**−0.11**	**0.01**
**6 Enjoyment**		4.33	0.81	0.907	**−0.14**	**−0.18**	**0.42**	**0.55**	**0.28**	**−**	**−0.26**	**0.64**	**0.30**	−0.02	**−0.09**	**−0.16**
**7 Stress**		6.09	3.54	0.751	**0.20**	**−0.28**	**−0.08**	**−0.21**	**−0.19**	**−0.17**	**−**	**−0.16**	−0.07	−0.05	0.07	**0.13**
**8 Intention to be Physically active**		4.05	0.85	0.813	**−0.14**	**−0.19**	**0.40**	**0.65**	**0.29**	**0.58**	**−0.16**	**−**	**0.44**	−0.04	**−0.14**	**−0.15**
**9 Physical activity**		50.35	48.57	–	**−0.07**	**−0.07**	**0.17**	**0.43**	**0.18**	**0.28**	**−0.06**	**0.43**	**−**	−0.06	**−0.11**	**−0.14**
**10 Age**		14.59	1.35	–	0.05	0.01	**−0.11**	**−0.10**	−0.03	**−0.16**	−0.03	**−0.10**	**−0.09**	−	**0.17**	**0.02**
**11 BMI**		20.89	4.37	–	**0.28**	**0.13**	−0.05	**−0.16**	**−0.13**	**−0.07**	**0.09**	**−0.10**	−0.02	**0.18**	**−**	**0.17**
**12 Sex**		–	–	–	**−0.07**	**−0.01**	**0.08**	**−0.19**	**−0.07**	**−0.08**	0.01	**−0.07**	**−0.15**	−0.03	**−0.07**	**−**

*Note.* Significant correlations appear in bold type.

**Table 2:** Measurement invariance analysis to ensure that the target items measure the same theoretical constructs (latent variables or factors) in all groups.

Measurement Invariance	χ^2^	*df*	RMSEA	SRMR	TLI	CFI	ΔCFI	ΔModel
Configural invariance	5138.057	1502	0.05	0.06	0.924	0.927	--	--
Weak factorial invariance	5253.286	1509	0.05	0.06	0.922	0.924	−0.003	2 vs. 1
Strong factorial invariance	5352.663	1511	0.05	0.06	0.920	0.922	−0.005	3 vs. 1
Strict factorial invariance	5869.539	11550	0.055	0.07	0.912	0.913	0.014	4 vs. 1

*Note.* χ^2^: Chi-square test; *df*: degrees of freedom; RMSEA: Root Mean Square Error of Approximation; SRMR: Standardized Root Mean Square Residual; TLI: Tucker–Lewis Index; CFI: Comparative Fit Index; ΔCFI: variations in CFI.
